# MED27 function is essential for cerebellar development and motor behaviour

**DOI:** 10.1093/brain/awaf237

**Published:** 2025-06-19

**Authors:** Sabrina Maher, Eloise Langlois Bernard, Charlotte Zaouter, Shunmoogum A Patten

**Affiliations:** Institut National de la Recherche Scientifique (INRS), Centre Armand Frappier Santé Biotechnologie, Laval, QC, Canada H7V 1B7; Département de Neurosciences, Université de Montréal, Montréal, QC, Canada H3C 3J7; Institut National de la Recherche Scientifique (INRS), Centre Armand Frappier Santé Biotechnologie, Laval, QC, Canada H7V 1B7; Département de Neurosciences, Université de Montréal, Montréal, QC, Canada H3C 3J7; Institut National de la Recherche Scientifique (INRS), Centre Armand Frappier Santé Biotechnologie, Laval, QC, Canada H7V 1B7; Institut National de la Recherche Scientifique (INRS), Centre Armand Frappier Santé Biotechnologie, Laval, QC, Canada H7V 1B7; Département de Neurosciences, Université de Montréal, Montréal, QC, Canada H3C 3J7

In the recent publication in *Brain* by Maroofian *et al.*,^1^ the authors provide a comprehensive clinical and radiological description of *MED27*-related disease. They particularly demonstrated that *MED27*-related disease manifests brain anomalies along with movement abnormalities. Several reports have also identified *MED27* variants in a neurodevelopmental disorder characterized by key features such mental retardation, cerebellar atrophy, spasticity, hypotonia and motor deficits.^2-4^ Wu *et al.*^3^ showed that homozygous missense variants in the gene *MED27* resulted in a significant reduction in MED27 protein expression. However, the pathogenicity of loss-of-function of MED27 and the mechanisms underlying neurodevelopmental defects in MED27-related disease currently remain largely unknown.

We investigated the role of MED27 in neurodevelopment using the zebrafish as a vertebrate model system. The zebrafish genome encodes a single *med27* orthologue that is highly conserved with human *MED27*. We generated a *med27* biallelic knockout (F0 KO) zebrafish using the CRISPR-Cas9 system.^5^ We found that *med27* F0 KO larvae exhibited a smaller body, eye and head size compared to wild-type control fish (Fig. 1A–D). In light of the neurological phenotypes of patients harbouring MED27 variants, we evaluated various aspects of the morphology and function of the CNS in *med27* F0 KO larvae. We investigated motor function in the *med27* F0 KO fish using the automated Noldus Ethovision XT behaviour monitoring system. We found that *med27* F0 KO exhibited significant motor impairments compared to controls (Fig. 1E and F). Haematoxylin and eosin (H&E) staining of transverse brain sections at 3 days post-fertilization (dpf) revealed structural differences and smaller brain size in *med27* F0 KO fish compared to wild-type larvae at 3 dpf and 5 dpf (Fig. 1G and H). Importantly, we observed a marked progressive loss of H&E staining in the cerebellum of *med27* F0 KO zebrafish brain compared to controls, suggesting a cellular depletion in the region (Fig. 1G). Taken together, these findings are in line with clinical observations in MED27 variant patients.

**Figure 1:**
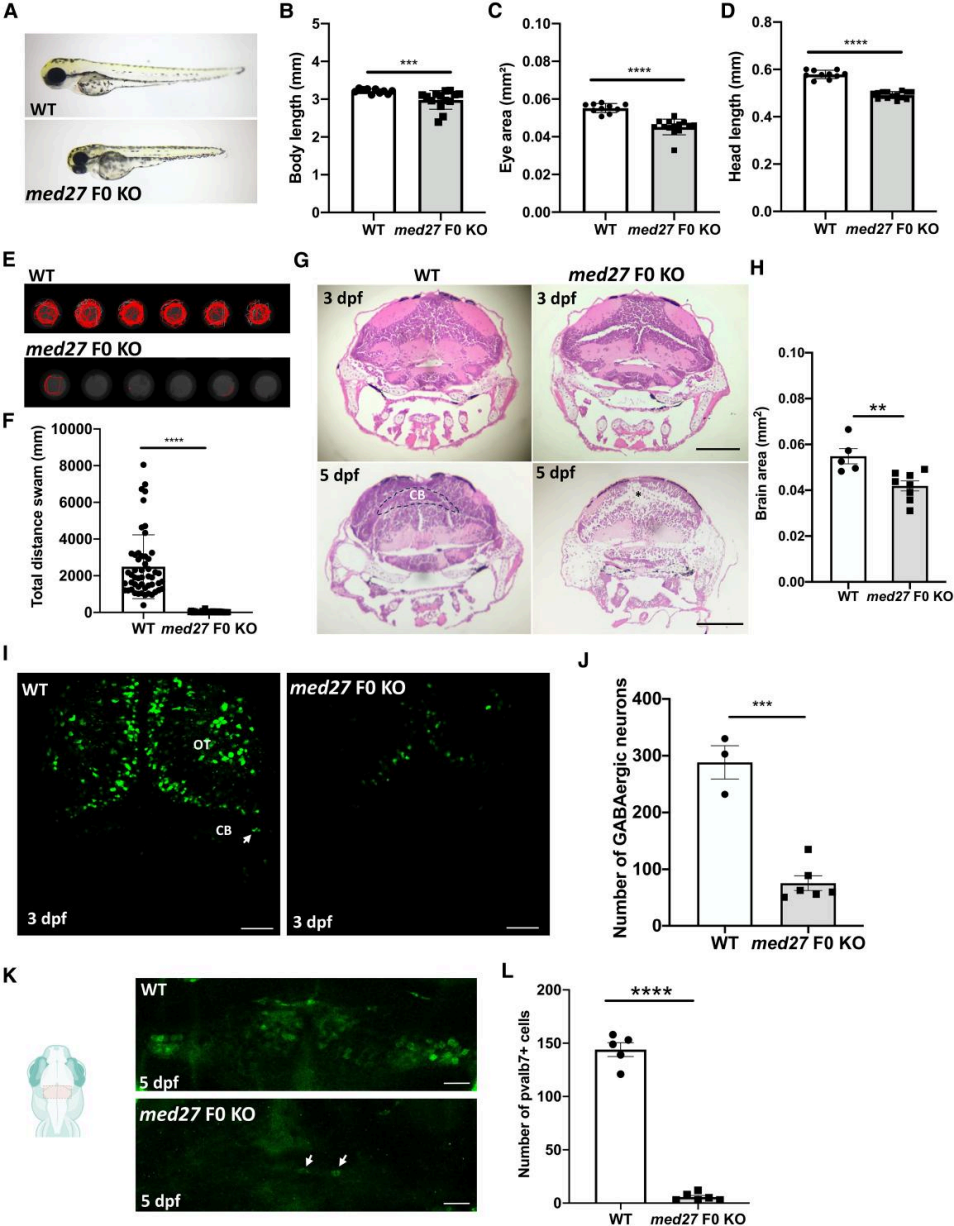
**Modelling *med27* knockout (KO) in zebrafish**. (**A**) Morphology of wild-type zebrafish (WT), and *med27* F0 KO larvae at 3 days post-fertilization (dpf). (**B**–**D**) Body length (**B**), head length (**C**) and eye area (**D**) of WT and *med27* F0 KO larvae at 3 dpf (*n* = 10–14). (**E**) Representative swimming tracks, and quantification of swimming and velocity of WT control and *med27* F0 KO fish at 4 dpf. (**F**) Quantification of total distance swam of *med27* F0 KO larvae (4 dpf, *n* = 48) compared to WT (4 dpf, *n* = 54) showed a significant reduction in locomotor behaviour. (**G**) Haematoxylin and eosin staining of midbrain brain sections of 3 and 5 dpf control and *med27 F0 KO* zebrafish. Black dashes demarcate the cerebellum region (CB) and an asterisk denotes major cell loss in the CB. Scale bar = 100 µm. (**H**) Quantification of brain size of *med27* F0 KO larvae (3 dpf, *n* = 5) compared to WT (3 dpf, *n* = 8) showed a significant reduction that could reflect microcephaly. (**I**) Representative images of GABAergic neurons (*dlx5a/6a*:GFP) in control and *med27* F0 KO zebrafish brain at 3 dpf. Scale bar = 50 µm. (**J**) Quantitative analysis demonstrated a significant decrease in the number of GABAergic neurons (*dlx5a/6a*:GFP) in *med27* F0 KO zebrafish (*n* = 6) compared to controls (*n* = 3). (**K**) Immunostaining of Purkinje cells (pvalb7) in WT and *med27* F0 KO zebrafish cerebellum at 5 dpf. Scale bar = 20 µm. (**L**) Quantitative analysis demonstrated a significant decrease in the number of Purkinje cells (pvalb7) in *med27* F0 KO zebrafish (*n* = 6) compared to WT control zebrafish (*n* = 5) cerebellum at 5 dpf. All data are represented as the mean ± standard error of the mean (SEM). Statistical significance was calculated by Student’s *t*-test or Mann–Whitney test, **P < 0.01; ***P < 0.001; ****P < 0.0001. *n* represents the number of fish.

Given the brain abnormalities in MED27 patients and *med27* F0 KO zebrafish, we next sought to examine the brain neuronal networks. We found a significant reduction in the number of GABAergic neurons in the *med27* F0 KO zebrafish brain at 5 dpf (Fig. 1I and J), including in the cerebellum. These findings align with the clinical evidence of the loss of GABAergic neurons in several neurodevelopmental disorders including mental retardation,^6^ a key feature in MED27 variant patients. To complement our observations of cerebellar abnormalities and GABAergic neuronal loss in *med27* F0 KO zebrafish, we assessed the cerebellar neuronal parvalbumin7 (pvalb7)-positive Purkinje cells which are important inhibitory GABAergic neurons for cerebellar function. We observed a significant reduction in the number of Purkinje cells within the cerebellum of *med27* F0 KO zebrafish brain (Fig. 1K and L). Overall, these data suggest that *med27* is involved in cerebellum development, particularly in populating GABAergic neuronal subtypes in vertebrates.

The zebrafish CNS proliferative profile is still very high at 2 dpf and is rapidly downregulated up to 5 dpf.^7^ In 5 dpf *med27* F0 KO brains, we strikingly found an increase in proliferating cells (Fig. 2A and B) and apoptosis (Fig. 2C and D), suggesting a failure in neurogenesis with defects in progenitor cells differentiating into GABAergic neurons. Interestingly, we also found a significant reduction in the nestin-positive neural stem cells (Fig. 2E and F), reinforcing that MED27 likely plays a role during early neurogenesis, particularly in regulating the development of early neural progenitors. To confirm this hypothesis, we compared the expression level of specific cerebellar neurogenesis markers^8^ (*atoh1a*, *atoh1b*, *atoh1c* and *ptf1a*, for Purkinje cell neurogenesis^9,10^). We found that the expression of all these markers is significantly decreased at 2 dpf in *med27* F0 KO larvae compared to controls by reverse transcription quantitative PCR (RT-qPCR) (Fig. 2G). These findings suggest that these changes caused by the loss of MED27 arise early on as of 2 dpf with important consequences on cerebellar neurogenesis at later developmental stages. Altogether, our data indicate an important role of MED27 in neurogenesis and cerebellar development.

**Figure 2:**
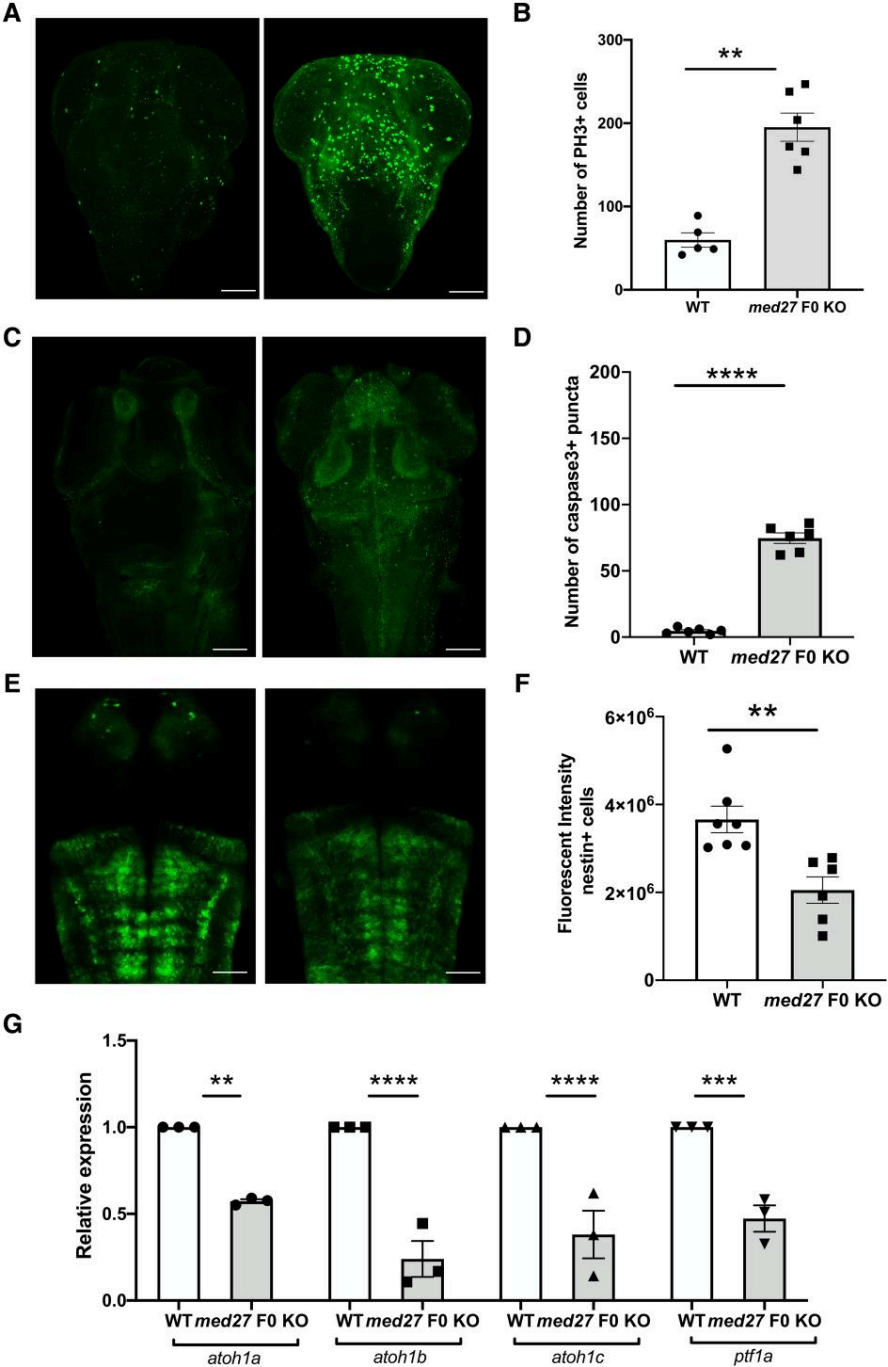
**Altered neurogenesis and increased apoptosis in zebrafish *med27* knockout (KO) brain**. (**A**) PH3 immunostaining in wild-type (WT) and *med27* F0 KO zebrafish brain at 3 days post-fertilization (dpf). Scale bar = 50 µm. (**B**) Quantitative analysis demonstrated a significant increase in the number of proliferative cells (PH3+) in *med27* F0 KO zebrafish (*n* = 6) compared to WT control zebrafish (*n* = 6) brain at 3 dpf. (**C**) Immunostaining for cleaved Caspase 3 in WT and *med27* F0 KO zebrafish brain at 3 dpf. Scale bar = 50 µm. (**B**) Quantitative analysis demonstrated a significant increase in the number of apoptotic cells (Caspase3+) in *med27* F0 KO zebrafish (*n* = 6) compared to WT control zebrafish (*n* = 6) brain at 3 dpf. (**E**) Representative images of neural progenitor cells (*nestin*:GFP) in WT and *med27* F0 KO zebrafish brain at 2 dpf. Scale bar = 50 µm. (**F**) Quantitative analysis demonstrated a significant decrease in the number of nestin-positive cells in *med27* F0 KO zebrafish (*n* = 6) compared to controls (*n* = 7). (**G**) Reverse transcription quantitative PCR of cerebellum neurogenesis markers (*atoh1a*, *atoh1b*, *atoh1c* and *ptf1a*) at 2 dpf in *med27* F0 KO zebrafish compared to controls.

## Supplementary Material

awaf237_Supplementary_Data

## Data Availability

The data providing the evidence of the study are available from the corresponding author upon reasonable request.
