# Supplementary material for: MED27 function is essential for cerebellar development and motor behaviour
Source: Brain. 2025 Jun 19;148(11):e100–3. doi: 10.1093/brain/awaf237 (PMC12588712; doi:10.1093/brain/awaf237)
Supplement: awaf237_Supplementary_Data [file awaf237_supplementary_data.pdf]

MED27 function is essential for cerebellar development and motor behaviour

Sabrina Maher, Eloise Langlois Bernard, Charlotte Zaouter, Shunmoogum A. Patten

Supplementary Data

Materials and Methods

Zebrafish husbandry

Wild-type (WT) *Danio rerio* (AB/TL strain) and transgenics (Tg) *GFAP*:GFP and *nestin*:GFP fish were maintained at 28°C at a light/dark cycle of 12/12 h in accordance with standard practices[1]. Embryos were raised at 28.5°C and collected and staged as previously described[2]. All experiments were performed in compliance with the guidelines of the Canadian Council for Animal Care and the local ethics committee of INRS.

Generation of med27 FO KO zebrafish model

Zebrafish *med27* F0 KO embryos were generated using CRISPR-Cas9 following an established genome editing protocol as previously described[3]. Three gRNAs targeting *med27* were selected, by choosing target sequences using Integrated DNA technologies (IDT; https://www.idtdna.com/site/order/designtool/index/CRISPR_PREDESIGN) design tools, with higher priority given to those with fewer off-target effects. Genotyping of *med27* mutant fish was performed by high-resolution melting (HRM) analysis and Sanger sequencing. Primers and sgRNAs are available upon request. Morphological analysis was performed under a Leica S6E stereoscope and analyzed using ImageJ Fiji Software (NIH).

Locomotor behavior

Zebrafish larvae at 4 dpf were individually transferred into a 96-well plate with 200µL of system water and locomotor activity was recorded for 30 min in the "light off" condition followed by 30 min in the "light on" condition. Data were analyzed by quantifying the total distance swam by

each larva (mm). Locomotor activity was recorded using the Basler GenIcam camera in the DanioVision recording chamber (Noldus) and data were collected using the Ethovision XT 12 software (Noldus).

H&E analysis of *med27* F0 KO zebrafish model

Zebrafish larvae at 3 and 5 dpf were fixed in 4% paraformaldehyde (PFA, Thermo Scientific) and embedded in paraffin. The larvae's brain was cut with a microtome in transverse section of 5 µm (Leica, RM 2155) and collected on slides. The slides were deparaffinized by 2 baths of xylene and rehydrated in a graded series of ethanol baths. They were then stained in hematoxylin and rinsed in water, briefly soaked in acid alcohol, air-dried, then put back in water for 1 min. They were dipped in lithium carbonate, air-dried, and rinsed in water again. The slides were then stained in eosin for and quickly dehydrated in 100% ethanol. They were cleared in xylene, mounted in "Quick-hardening mounting medium" (Sigma) and were left to dry overnight. The stained histological slides were imaged under a microscope (Leica S6E) and analysis of brain sections was performed using the Fiji/Image J (NIH) Imaging software.

Apoptosis analysis

To prevent pigmentation, zebrafish embryos of 10-24 hpf were treated with 0.003% PTU (Sigma). Zebrafish larvae (5 dpf) were fixed in 4% PFA overnight at 4°C, were washed in PBS-Triton 0.1% (PBST, Sigma), permeabilized 2h in PBST 1% and blocked 1 hour in blocking buffer (2% Normal Goat Serum (NGS), 1% DMSO, 1% Triton in PBS) (Sigma-Aldrich). They were incubated with the cleaved caspase 3 antibody overnight at 4°C (1:200, Cell Signaling #9661S), washed in 0.1% PBST and incubated with the secondary antibody Alexa Fluor 488 goat anti-rabbit (1:1000,

Invitrogen #A-11008) overnight at 4°C. Larvae were then washed in 0.1% PBST, mounted ventrally on slides in fluoromount (ThermoFisher) and imaged with a confocal microscope (Zeiss, LSM 780).

Neuronal network

To image and quantify GABAergic neurons (*dlx5a/6a*:GFP) in 3 dpf zebrafish larvae of Tg (*dlx5a/6a*:GFP::WT) and Tg (*dlx5a/6a*:GFP::*med27* KO zebrafish larvae were fixed in 4% PFA for 20 mins. To image and quantify neural progenitor cells, *nestin*:GFP was used. Zebrafish larvae were mounted in 0.5% low-melting point agarose (Invitrogen; 16520-100). Z-stack images were taken using a Zeiss LSM780 confocal microscope (Carl Zeiss, Germany). GABAergic neurons (*dlx5a/6a*:GFP) were counted in WT and *med27* F0 KO zebrafish manually and blindly using the Cell Counter plug-in for Fiji/Image J (NIH) Imaging software.

Whole-mount immunostaining

Zebrafish larvae were fixed overnight at 4°C in 4% paraformaldehyde prepared in phosphate-buffered saline (PBS). After fixation, the samples were rinsed three times for 15 minutes each at room temperature in PBS containing 0.1% Tween-20 (PBS-Tween) and then washed twice for 10 minutes each in PBS-DT (1% bovine serum albumin (BSA), 1% Triton, and 1% dimethyl sulfoxide (DMSO)). Then larvae were incubated for 1 hour in a blocking solution composed of 5% normal goat serum (NGS), 1% BSA, 1% DMSO, and PBS-Triton (1%). Primary antibodies: anti-pvalb7 (1:200; Millipore Sigma, MAB1572) and anti-PH3 (1:250; Millipore Sigma, Cat# 06–570) were then added to the blocking solution and incubated overnight at 4°C. On the following day, after three washes of 15 minutes each in PBS-Tween, the larvae were incubated with a secondary antibody, goat anti-rabbit or goat anti-mouse Alexa Fluor 488 (Invitrogen), diluted in blocking solution, and maintained at 4°C overnight. Finally, after an additional three 15-minute washes in PBS-Tween at room temperature the next day, the larvae were mounted in Fluoromount-G (Invitrogen, 00–4958-02) and imaged using Zeiss LSM 780 confocal microscope (Carl Zeiss, Germany). Quantification was performed using the Cell Counter plug-in for Fiji/Image J (NIH) Imaging software.

Quantitative reverse transcription polymerase chain reaction (qRT-PCR)

Total RNA was isolated from 2 dpf zebrafish embyo (N = 3, n = 20) using TriReagent and 1 µg of RNA was used for cDNA synthesis using cDNA vilo kit (ThermoFisher). qRT-PCR was performed with SYBR Green mix (BIORAD) with a Lightcycler96® (Roche). Gene expression was analyzed relative to the housekeeping gene elf1α. Primers used for *atoh1a, atoh1b, atoh1c* and *ptf1a* are available upon request.

Statistical analysis

All statistical analyses were performed and graphs were plotted using the Graphpad PRISM software. Significance is indicated as ** $p<0.01$, *** $p<0.001$ and *** $p<0.0001$. n represents number of fish. Data are presented as Mean±SEM.

1. Westerfield M. *The zebrafish book : a guide for the laboratory use of zebrafish (Brachydanio rerio)*. M. Westerfield; 1993.
2. Kimmel CB, Ballard WW, Kimmel SR, Ullmann B, Schilling TF. Stages of embryonic development of the zebrafish. *Dev Dyn*. Jul 1995;203(3):253-310. doi:10.1002/aja.1002030302
3. De Pace R, Maroofian R, Paimboeuf A*, et al*. Biallelic BORCS8 variants cause an infantile-onset neurodegenerative disorder with altered lysosome dynamics. *Brain*. May 3 2024;147(5):1751-1767. doi:10.1093/brain/awad427
